# Outcomes of a randomized controlled trial of nutrition education to promote farmers’ market fruit and vegetable purchases and consumption among women enrolled in the Special Supplemental Nutrition Program for Women, Infants, and Children (WIC)

**DOI:** 10.1186/s40795-017-0172-0

**Published:** 2017-06-21

**Authors:** Jennifer Di Noia, Dorothy Monica, Alla Sikorskii, Karen Weber Cullen

**Affiliations:** 1 Department of Sociology, William Paterson University, 300 Pompton Road, Wayne, NJ 07460 USA; 2 Saint Joseph’s WIC Program, 185 6th Avenue, Paterson, NJ 07524 USA; 3 College of Nursing, University of Arizona, 1305 North Martin Avenue, Room 419, PO Box 210203, Tucson, AZ 85721 USA; 4 USDA/ARS Children’s Nutrition Research Center, Department of Pediatrics, Baylor College of Medicine, 1 Baylor Plaza, Houston, TX 77030 USA

**Keywords:** Randomized controlled trial, Farmers’ market, Farmers’ market nutrition program, Cash value voucher, Fruits, Vegetables, Income, WIC program, Internet

## Abstract

**Background:**

The Special Supplemental Nutrition Program for Women, Infants, and Children (WIC) provides participants seasonal Farmers’ Market Nutrition Program (FMNP) vouchers to purchase fruits and vegetables (FV) at farmers’ markets and monthly cash value vouchers (CVV) redeemable at farmers’ markets. Despite the promise of FMNP vouchers and CVV for improving FV access among WIC participants, voucher redemption rates are low. This study evaluated WIC Fresh Start (WFS), a theory-driven, web-based lesson to promote FV intake, the redemption of CVV at farmers’ markets, FMNP voucher redemption, and farmers’ market-related knowledge, attitudes, and skills among women enrolled in WIC.

**Methods:**

The lesson was evaluated in a four-arm randomized controlled trial. The setting was a large New Jersey-based WIC agency located in a densely populated, urban area. Participants (*N* = 744) were stratified based on FMNP voucher receipt and randomized to receive the WFS lesson or WIC online existing health education. Lesson effects on targeted outcomes were examined at posttest (2 weeks after the lesson) and 3 and 6 months after posttesting.

**Results:**

Receipt of the WFS lesson was associated with FMNP voucher redemption (in the subset of participants preferring to speak Spanish); improvements in knowledge of the FMNP, locally grown seasonal items, seasonal items found at farmers’ markets in July, WIC-authorized farmers’ markets and food- and farmers’ market-specific knowledge; ever having purchased and intentions to purchase FV at a farmers’ market; FV food safety and preparation skills; and modest gains in the redemption of CVV at farmers’ markets. FV intake did not differ over time by trial arm.

**Conclusions:**

Findings aid understanding of effective approaches to promote farmers’ market use and farmers’ market-related knowledge and skills among WIC participants. Further research is needed to explore factors that may explain the lack of lesson effects on FV intake.

**Trial registration:**

NCT02565706

## Background

The Special Supplemental Nutrition Program for Women, Infants, and Children (WIC) is designed to safeguard the health of low-income women, infants, and children up to age 5 who are nutritionally at risk by providing healthy foods to supplement diets (via WIC food packages), nutrition education, and counseling and referrals to health and other social services [1]. Food packages are provided to specific groups of WIC participants (e.g., pregnant and breastfeeding women, infants, and children) [2]. The packages were revised in 2009 to include the addition of a monthly cash value voucher (CVV) for fruit and vegetable (FV) purchases (vouchers were valued at $10 for women and $6 for children) [2]. The revisions were based, in part, on the recommendations of an Institute of Medicine report on the nutritional status of the WIC population that found lower than recommended intakes of FV among women and lower than recommended intakes of vegetables among children aged 2 to 4 years [2, 3]. States had the option to authorize the redemption of CVV at farmers’ markets. In in 2009, 13 states and the District of Columbia adopted this policy [2]. Prior to the revisions, the only FV subsidy available to WIC participants was provided through the WIC Farmers’ Market Nutrition Program (FMNP) [4].

The FMNP provides WIC participants up to $30 in seasonal vouchers to purchase FV from WIC-authorized farmers at farmers’ markets [5]. The literature indicates that those who receive FMNP vouchers have higher vegetable intake and higher FV intake combined relative to those who do not [6–8]. Farmers’ market use is greater among participants who previously redeemed FMNP vouchers as compared to those who did not receive or redeem them [9]. Relative to vouchers alone, stronger FMNP effects on FV intake are found when vouchers are supplemented with nutrition education [10].

Despite the promise of the FMNP, nationwide, the FMNP voucher redemption rate was 59% in 2014 [11]. The CVV redemption rate at farmers’ markets is less than 1% among states reporting this information [12]. Whereas FMNP vouchers are issued seasonally, not every family receives them due to funding constraints [4]. CVV are issued monthly to all WIC participants [4]. As such, CVV afford participants ongoing opportunities to purchase FV at farmers’ markets during the farmers’ market season in states authorizing their redemption at these venues.

FV subsidies provided by WIC have the potential to improve FV availability and intake in this population [13]. Yet, little is known about effective approaches to encourage farmers’ market use in this population. This report describes the outcome evaluation of WIC Fresh Start (WFS), a theory-driven, web-based lesson to promote farmers’ market FV purchases and consumption among women enrolled in WIC [14]. The research was a collaboration between a university-based researcher and New Jersey state and local WIC agency representatives. New Jersey is among the states authorizing the redemption of CVV at farmers’ markets.

### Research objective

Evaluate the effects of the WFS lesson relative to WIC online existing health education (EHE) on primary (FV intake, FMNP voucher redemption, and the redemption of CVV at farmers’ markets) and secondary (farmers’ market-related knowledge, attitudes, and skills) outcomes immediately following and 3- and 6-months after the lesson in a stratified four-arm design with FMNP voucher receipt as the stratification factor (WFS, WFS plus FMNP vouchers [WFSV], EHE, and EHE plus FMNP vouchers [EHEV]). An exploratory research objective was to determine whether the effects of arm and lesson on primary outcomes were moderated by participant characteristics.

### Study hypotheses

1. Women in the WFSV arm will have higher FV intake and better secondary outcomes at posttest and follow-up measurements relative to women in the three other arms.

2. Women who receive the WFS lesson will have higher FV intake and better secondary outcomes at posttest and follow-up measurements relative to women who receive EHE.

3. The redemption of CVV at farmers’ markets will be higher among women who receive the WFS lesson relative to women who receive EHE.

4. FMNP voucher redemption will be higher among FMNP voucher recipients who receive the WFS lesson relative to those who receive EHE.

## Methods

### Design

The lesson was evaluated in a double-blinded, four-arm, randomized controlled trial. Participants were stratified based on FMNP voucher receipt (at the time of the study, pregnant and breastfeeding women and children aged 2 to 5 years were eligible to receive vouchers), orally administered a pretest, and randomized to receive the WFS lesson or EHE (any of 7 lessons of their choosing as described below). Two weeks after the lesson, participants were contacted by telephone to complete the posttest. Telephone-administered follow-up assessments were conducted 3 and 6 months after the posttest.

### Setting and sample

The setting was a large New Jersey-based WIC agency located in a densely populated, urban area. All study data were collected at this location. Inclusion criteria were being an English- or Spanish-speaking pregnant or postpartum WIC participant or female caregiver of an infant or child participant served by the collaborating WIC agency. In New Jersey, WIC participants are required to complete two nutrition education contacts per certification period (every 6 months). Participants have the option to complete an in-person individual or group lesson or an online lesson (during the WFS trial, 7 online lessons were available [topics were iron, breastfeeding, being active, FV, calcium, cholesterol, and oral health]). To equalize attention and mode of lesson delivery between trial arms, women also had to be willing to complete an online lesson. Exclusion criteria were having known restrictions on food intake and being classified as high-risk by WIC (defined based on an extensive list of statewide risk criteria, e.g., being anemic or having a body mass index ≥30) [15]. High-risk women were excluded because they are required to receive in-person nutrition education to satisfy their nutrition education requirement [15].

Trained bi-lingual (English/Spanish) research assistants screened women for eligibility when presenting for services. Eligible women received oral and written descriptions of the study. Of 1345 women who were approached, 64 were ineligible, 537 were eligible but declined to participate, and 744 were enrolled. The sample was sufficiently large to detect a .60 serving/day difference in FV intake in pairwise posttest comparisons by arm (standardized effect size = .41), and an effect size of .26 or greater in longitudinal analyses. The study was approved by the William Paterson University Institutional Review Board for Human Subject Research (2014–368) and registered with ClinicalTrials.gov (NCT02565706). It was conducted and is being reported in accordance with the Consolidated Standards of Reporting Trials (CONSORT) guidelines [16]. Participants were enrolled between June 1, 2015 and August 12, 2015. Posttest and follow-up assessments were conducted between June 15, 2015 and March 2, 2016. All women provided informed written consent prior to their study involvement.

### Randomization sequence generation, allocation concealment, and blinding

Enrollment forms with allocation information (concealed by a cover page) were developed using a computer-generated binary randomization sequence (created by AS). The research assistant who enrolled the participant added her name to the form and assigned her to a second research assistant who met with the participant in a 1:1 session to obtain the participant’s informed consent, orally administer the pretest, and assist the participant in accessing an online lesson. The enrolling research assistant informed the second research assistant of the allocation (by removing the cover page) at the start of the 1:1 session. Although research staff and participants were aware that the research was evaluating outcomes of WIC online health education, they did not know which lesson (WFS or any of the seven EHE lessons) was the experimental lesson.

### Online lessons

#### WFS lesson

WFS consisted of an online lesson with handouts and follow-on content delivered one, two, and three months after the lesson. The theoretical underpinnings of the lesson and the included content are described elsewhere [14]. Briefly, WFS comprised three modules, each consisting of 1) behavior change content delivered via a short video segment and audio output to maximize accessibility for low-literate learners (videos were approximately 3 min in length) [17], and 2) an interactive activity to build targeted knowledge, attitudes, and skills. Participants could complete the lesson in 10–15 min, depending on how long it took them to complete the interactive activities. To enhance the credibility and relevance of messages, videos featured WIC participants [18]. Follow-on content was delivered via videos featuring women from the lesson (videos were 4–5 min in length) sent via emails containing links to the videos.

#### EHE

EHE lessons consisted of an introductory segment presented with online text and graphics and four lesson activities. The activities were designed to enable women to read further on the topic and to reinforce key points of the lesson. Because WIC participants are required to complete one of the four activities for the lesson to satisfy their WIC nutrition education requirement, for the trial, they were also required to complete one activity (of their choosing) after completing the introductory segment. Lessons required 3–5 min to complete, depending on the participants’ reading speed. Although a FV lesson was included, the topics addressed in the lesson (i.e., health benefits of eating more FV, recommended amounts of FV to consume daily, and vitamins found in FV) differed from those addressed in the WFS lesson. All lessons were available in English and Spanish. The WFS videos were filmed twice (once with English-speaking and once with Spanish-speaking WIC participant-narrators).

### Measures

Research assistants orally administered study measures in English or Spanish (depending upon the participant’s preference) at each measurement occasion. To enhance the quality of measurements, research assistants were trained on data collection procedures in a full day training prior to their entry to the field. Throughout the trial, the lead investigator convened daily debriefing sessions with research staff to review data collection procedures and to discuss problems, if any, encountered during data collection and strategies to avoid or resolve them.

### Primary outcome measures

#### FV intake

Two dimensions of FV intake were assessed with validated instruments: the frequency of intake (times per day FV [including FV juices] were consumed) and the quantity of intake (cups per day of FV [including FV juices] consumed) [19–21]. The reference period was the past 2 weeks. To enhance the accuracy of reporting, at pretest, participants were shown an 8-oz measuring cup and 12-oz glass with 8-oz fill line marked when reporting amounts consumed of foods and juices and were asked to recall these portion size estimation aids at subsequent measurements.

#### Voucher redemption

In New Jersey, FMNP vouchers are issued annually and can be redeemed between June 1 and November 30. The local WIC agency provided data on FMNP vouchers issued to participants in 2015 and CVV issued between June 1 and November 30. The state WIC agency provided data on all vouchers redeemed by participants between June and November.

### Secondary outcome measures

Measures of farmers’ market related knowledge (knowledge of the FMNP; markets with WIC-authorized farmers [and correspondingly, the location, hours of operation, and time of year markets were open]; locally grown, seasonal FV; FV found at farmers’ markets in July; and the selection, storage, and parts eaten of seasonal FV), attitudes (attitudes toward FV sold at farmers’ markets and positive outcome expectations for consuming locally grown FV) and skills (FV food safety skills and skills in preparing locally grown seasonal FV) were developed for the research and validated using data collected at pretest. Participants also reported their lifetime and recent (past two weeks) farmers’ market use and intentions to purchase FV at a farmers’ market in the next two weeks. Farmers’ market asking skills (whether participants asked farmers about how to store FV, how to prepare FV, and items that were unfamiliar to them) were assessed among those who reported having purchased FV at a farmers’ market in the past two weeks. Scores on multi-item measures were computed as the sum of item responses (for true/false items, correct responses were summed). Information on the measures (items per measure, illustrative items, response formats, score ranges, and reliability coefficients) is presented in Table 1.

**Table 1:** Secondary outcome measures administered in the WIC Fresh Start trial

Measure (number of items), *illustrative item*, and [response format]	Score range	Reliability coefficient
Index of Knowledge of the Farmers’ Market Nutrition Program (6) *Not all farmers at farmers’ markets accept farmers’ market vouchers*. [true/false]	0–6	NA
Knowledge of WIC-authorized Farmers’ Markets (1) *Do you know of a farmers’ market near you where the farmers accept WIC farmers’ market and cash value vouchers*? [yes/no]	0–1	NA
Farmers’ Market-specific Knowledge (2) *Do you know what time of year* [area market] *is open?* [yes/no]	0–2	.67
Familiarity with Locally Grown Seasonal Items (3) *Do you know what kale is?* [yes/no]	0–3	.69
Knowledge of Locally Grown Seasonal FV found at Farmers’ Markets in July (9) *Likely to find yellow summer squash at farmers’ markets in the month of July?* [yes/no]	0–9	.63
Food-specific Knowledge (13) *Storing blueberries on the same shelf as vegetables is not recommended because they give off a gas that will make the vegetables age quicker.* [true/false]	2–13	.51
Attitudes towards Farmers’ Market FV (6) *I don’t like the way farmers’ market FV look*. [7-point Likert scale; 1 = not at all to 7 = very much]	6–42	.84
Positive Outcome Expectations for Consuming Locally Grown FV (3) *Locally grown FV are full of flavor.* [yes/no]	0–3	.42
FV Food Safety Skills (13) *Refrigerate FV that are precut or peeled.* [7-point Likert scale; 1 = never to 7 = always]	19–85	.70
Farmers’ Market FV Preparation Skills (12) *How would you rate your skill in preparing cucumbers?* [7-point Likert scale; 1 = definitely could not make to 7 = definitely could make]	12–84	.81
Lifetime Farmers’ Market FV Purchases *Have you ever purchased FV at a farmers’ market?* [yes/no]	0–1	NA
Recent Farmers’ Market FV Purchases *Have you purchased FV at a farmers’ market in the past 2 weeks?* [yes/no]	0–1	NA
Farmers’ Market Asking Skills (3) *During this trip to the market, did you ask farmers how to store FV?* [yes/no]	0–3	.65
Intentions to Purchase FV at a Farmers’ Market *Do you intend to purchase FV at a farmers’ market in the next 2 weeks?* [yes/no]	0–1	NA

WIC indicates the Special Supplemental Nutrition Program for Women, Infants, and Children; *FV* fruit and vegetable(s), *NA* not applicable. Reliability assessed with Cronbach’s α and Kuder-Richardson 20 coefficients (for dichotomous indicators). For the Index of Knowledge of the Farmers’ Market Nutrition Program, reliability was not assessed because this construct is variable (implementation of the Farmers’ Market Nutrition Program varies by state; for example, states may differ in the number and face value of vouchers issued). As such, developing a stable scale is not possible and psychometrics do not apply. Farmers’ market asking skills were assessed among participants having reported shopping at a farmers’ market in the past two weeks

### Participant characteristics

At baseline, participants reported their age; pregnancy, breastfeeding, marital, and employment status; race/ethnicity; origin; nativity; country of birth (foreign-born); preferred language; language(s) spoken at home; educational attainment; educational attainment of spouse or partner; number of children and other adults in the household; and participation in assistance programs and completed validated measures of food security status and social desirability trait [22, 23].

### Implementation measures

Implementation measures assessed at baseline included lesson dose (data on the number of lesson modules and activities participants completed [a total of three each for the WFS lesson and one each for EHE]), EHE lessons, if any, completed prior to the study, and among those randomized to receive EHE, the EHE lesson completed during the trial. At 3-month follow-up, participants reported whether they opened follow-up emails and watched videos sent.

### Data analyses

#### Outcome analyses

Using an intent-to-treat approach, hypotheses 1 and 2 were tested using linear mixed-effects models with 3 repeated measures: posttest and 3- and 6-month follow-up. Covariates included baseline measures of each outcome and the following prognostic factors (potential influences on FV intake): pregnancy status, breastfeeding status, and receiving assistance other than WIC and social desirability trait (to minimize its potentially biasing effects on self-reported FV intake) [24–27]. Time was entered in the models as a categorical variable to model potentially non-linear patterns in outcomes over time. A time by arm interaction term was included to assess potentially changing intervention effects as time progressed. Least square means and standard errors were obtained for each level of the interaction term. In addition to the four-level arm variable, a two-level variable of the lesson received (WFS or EHE) was evaluated in separate models. T-tests of pairwise differences between least square means specified in the hypotheses were used to determine immediate lesson effects and whether observed effects were sustained over time.

Logistic regression analysis was used to relate voucher redemption (yes/no) to the lesson received (hypotheses 3 and 4) and covariates. Preliminary analyses revealed that the proportion of participants redeeming CVV at farmers’ markets was small. Differences by lesson in CVV redemption were therefore examined with cross tabulations and Fisher’s exact test.

#### Exploratory moderator analyses

To explore moderation, condition by moderator interaction terms were added to the aforementioned models examining the effects of arm and lesson on FV intake and FMNP voucher redemption. Included as potential moderators were prognostic factors, social desirability trait, and variables hypothesized to influence the relationship between lesson exposure and changes in primary outcomes, i.e., Hispanic ethnicity; foreign-born, employment, and food security status; language preference; educational attainment; and having children aged 2 to 5 years. For each outcome, least square means were compared by condition across levels of the potential moderator. All analyses were conducted with SAS 9.4, 2013, SAS Institute Inc., Cary, NC, USA. For tests of statistical significance, α = .05.

## Results

**Fig. 1:**
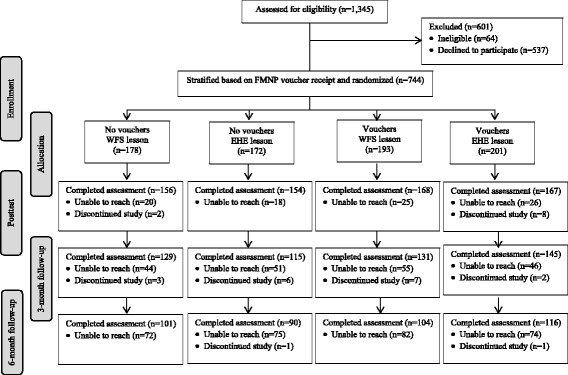
Flow of participants through the trial. WIC indicates Special Supplemental Nutrition Program for Women, Infants, and Children; FMNP, Farmers’ Market Nutrition Program; WFS, WIC Fresh Start, EHE, online existing health education

Of the 744 participants, 394 received FMNP vouchers and 350 did not; 371 received the WFS lesson and 373 received EHE. A small number of women (*n* = 30) discontinued their study involvement. The flow of participants through the trial is shown in the Fig. 1.

Participants had a mean (SD) age of 28.97 (6.83) years; 17% were pregnant and 21% were breastfeeding. Most were born in the U.S. (60%) and were Hispanic (59%; primarily of Dominican and Puerto Rican origin), with a high school education or less (50%), and lived with, on average, two children and one other adult. Although most (71%) reported English as their preferred language, 65% of foreign-born participants reported Spanish as their preferred language.

At baseline, participants reported consuming fruits and vegetables on average, 2.32 (SD = 1.91) and 2.16 (SD = 1.59) times/day, respectively. Fruits were reported to have been consumed more often than fruit juices (mean = 1.47, SD = 1.23 and mean = .86, SD = 1.11 times/day, respectively). Participants reported consuming greater amounts of fruits than vegetables (mean = 2.73, SD = 1.80 and mean = 1.38, SD = 1.16 cups/day respectively).

Ninety-four percent of participants completed all modules and activities in the lesson they received. Small and similar percentages of participants across arms (9%–16%) completed EHE lessons prior to the study (most commonly, lessons on FV and breastfeeding). Most women randomized to receive EHE during the trial chose to complete lessons on FV (29%), breastfeeding (22%), and being active (18%). Among those who received the WFS lesson, 302 (81%) provided an email address at sign-on. Of these 302 women, 33%, 16%, and 11%, respectively, reported opening emails sent one, two, and three months after the lesson. Participant characteristics and EHE lessons completed before and during the study by trial arm are shown in Table 2.

**Table 2:** Participant characteristics and EHE lessons completed before and during the study by trial arm

	Trial arm				
	WFS	WFSV	EHE	EHEV	Total
Age, mean (SD)	28.83 (7.31)	29.25 (6.44)	28.89 (6.56)	28.91 (7.03)	28.97 (6.83)
Pregnancy status					
Not pregnant	162 (91%)	145 (75%)	151 (88%)	157 (78%)	615 (83%)
Pregnant	16 (9%)	48 (25%)	21 (12%)	44 (22%)	129 (17%)
Breastfeeding status					
Not breastfeeding	149 (84%)	139 (72%)	147 (85%)	150 (75%)	585 (79%)
Breastfeeding	29 (16%)	54 (28%)	25 (15%)	51 (25%)	159 (21%)
Race/ethnicity					
African American	59 (34%)	47 (25%)	47 (28%)	68 (34%)	221 (30%)
Hispanic	103 (59%)	124 (64%)	99 (57%)	110 (55%)	436 (59%)
White or other	9 (5%)	17 (9%)	22 (13%)	18 (9%)	66 (9%)
Two or more races	4 (2%)	3 (2%)	3 (2%)	4 (2%)	14 (2%)
US-born					
No	73 (41%)	81 (42%)	60 (35%)	84 (42%)	298 (40%)
Yes	105 (59%)	112 (58%)	112 (65%)	117 (58%)	446 (60%)
Preferred language					
English	126 (70%)	131 (68%)	130 (76%)	138 (69%)	525 (71%)
Spanish	49 (28%)	62 (32%)	41 (23%)	59 (29%)	211 (28%)
Other	3 (2%)	--	1 (1%)	4 (2%)	8 (1%)
Marital status					
Married	36 (20%)	48 (25%)	44 (26%)	43 (21%)	171 (23%)
Separated	15 (8%)	23 (12%)	21 (12%)	23 (11%)	82 (11%)
Widowed	--	--	--	1 (1%)	1 (1%)
Never married	81 (46%)	81 (42%)	65 (37%)	82 (41%)	309 (41%)
Divorced	14 (8%)	6 (3%)	10 (6%)	5 (3%)	35 (5%)
Living with partner	32 (18%)	35 (18%)	32 (19%)	47 (23%)	146 (19%)
Educational attainment					
Less than high school education	33 (19%)	35 (18%)	31 (18%)	36 (18%)	135 (18%)
High school diploma or equivalent	59 (33%)	59 (31%)	57 (33%)	64 (32%)	239 (32%)
More than a high school education	86 (48%)	99 (51%)	84 (49%)	101 (50%)	370 (50%)
Educational attainment of spouse or partner					
Less than high school education	25 (18%)	30 (21%)	34 (26%)	29 (18%)	118 (21%)
High school diploma or equivalent	67 (49%)	59 (42%)	55 (42%)	90 (55%)	271 (47%)
More than a high school education	45 (33%)	53 (37%)	42 (32%)	45 (27%)	185 (32%)
Number of children in household under 19 years of age, mean (SD)	2.04 (1.29)	2.21 (1.22)	2.06 (1.38)	2.19 (1.20)	2.13 (1.27)
Number of other adults in household, mean (SD)	1.15 (1.08)	.98 (1.05)	1.19 (1.11)	1.19 (.99)	1.12 (1.06)
Employment status					
Employed	76 (43%)	64 (33%)	76 (44%)	70 (35%)	286 (38%)
Unemployed	102 (57%)	129 (67%)	96 (56%)	130 (65%)	457 (62%)
Receiving assistance other than WIC					
No	48 (27%)	54 (28%)	69 (40%)	55 (27%)	226 (30%)
Yes	130 (73%)	139 (72%)	103 (60%)	146 (73%)	518 (70%)
Food insecure					
No	69 (39%)	96 (50%)	80 (47%)	92 (46%)	337 (45%)
Yes	109 (61%)	97 (50%)	92 (53%)	109 (54%)	407 (55%)
Social desirability trait, mean (SD)	7.94 (1.69)	7.74 (1.59)	7.66 (1.69)	7.66 (1.70)	7.74 (1.66)
Previously completed EHE lesson					
No	160 (90%)	162 (84%)	156 (91%)	175 (87%)	653 (88%)
Yes	18 (10%)	31 (16%)	16 (9%)	26 (13%)	91 (12%)
EHE lesson completed during trial					
FV			59 (35%)	48 (24%)	107 (29%)
Breastfeeding			28 (16%)	54 (27%)	82 (22%)
Being active			33 (19%)	34 (17%)	67 (18%)
Oral health			16 (10%)	22 (10%)	38 (10%)
Cholesterol			12 (7%)	16 (8%)	28 (8%)
Iron			11 (6%)	17 (9%)	28 (8%)
Calcium			12 (7%)	10 (5%)	22 (5%)

WIC indicates the Special Supplemental Nutrition Program for Women, Infants, and Children; *WFS* WIC Fresh Start, *WFSV* WIC Fresh Start plus Farmers’ Market Nutrition Program vouchers; *EHE* online existing health education, *EHEV* online existing health education plus Farmers’ Market Nutrition Program vouchers. All values are reported as n (%) unless otherwise noted

### Lesson effects on FV intake and secondary outcomes (hypotheses 1 and 2)

FV intake did not differ over time by trial arm or lesson (data not in tables). As shown in Table 3, significant differences by arm (favoring those in the WFS arm relative to the three other arms) were found in knowledge of the FMNP at posttest (*p* < .0001) and 3- and 6-month follow-up (*p* = .0044 and .0092, respectively), FV food safety skills at posttest (*p* < .0001) and 3- and 6-month follow-up (*p* = .0010 and .0359, respectively), farmers’ market-specific knowledge at posttest (*p* = .0329) and 3-month follow-up (*p* = .0293), familiarity with locally grown seasonal items at 6-month follow-up (*p* = .0361), knowledge of seasonal items found at farmers’ markets in July at posttest (*p* = .0195), food-specific knowledge at posttest (*p* < .0001) and 3- and 6-month follow-up (*p* = .0001 and .0397), and farmers’ market FV preparation skills at posttest (*p* = .0421).

**Table 3:** Least square means and standard errors for secondary outcomes over time by trial arm

	Least square means and standard errors											
	Posttest				3-month follow-up				6-month follow-up			
	WFS	WFSV	EHE	EHEV	WFS	WFSV	EHE	EHEV	WFS	WFSV	EHE	EHEV
Knowledge of the FMNP	**4.88 (.08)**	**5.07 (.07)**	**4.45 (.08)**	**4.85 (.07)**	**4.79 (.09)**	**5.07 (.08)**	**4.71 (.09)**	**5.01 (.08)**	**4.67 (.10)**	**5.11 (.09)**	**4.82 (.10)**	**4.90 (.09)**
Attitudes towards FMFV	12.27 (.66)	11.05 (.59)	10.70 (.65)	11.10 (.60)	11.92 (.70)	10.24 (.65)	11.48 (.73)	11.42 (.63)	12.03 (.78)	10.19 (.73)	11.45 (.81)	12.27 (.70)
FV food safety skills	**67.80 (.92)**	**68.47 (.82)**	**63.99 (.91)**	**63.49 (.84)**	**66.70 (.98)**	**66.58 (.91)**	**64.13 (1.01)**	**62.59 (.88)**	**69.11 (1.10)**	**66.44 (1.02)**	**66.13 (1.13)**	**65.23 (.98)**
FM-specific knowledge	**1.37 (.08)**	**1.65 (.07)**	**1.39 (.09)**	**1.51 (.08)**	**1.42 (.09)**	**1.75 (.08)**	**1.51 (.10)**	**1.63 (.08)**	**1.51 (.10)**	1.69 (.09)	1.35 (.11)	1.68 (.09)
FM asking skills	.55 (.33)	.92 (.25)	.45 (.32)	1.14 (.28)	.49 (.35)	.85 (.33)	1.31 (.56)	.68 (.34)	1.29 (.40)	1.31 (.45)	1.30 (.59)	1.66 (.46)
Familiarity with LGSI	2.81 (.05)	2.82 (.04)	2.70 (.05)	2.67 (.04)	2.79 (.05)	2.86 (.05)	2.76 (.05)	2.75 (.05)	**2.81 (.06)**	**2.93 (.05)**	**2.80 (.06)**	**2.70 (.05)**
Knowledge of LGSI found at FM in July	**8.08 (.11)**	**8.22 (.11)**	**7.99 (.11)**	**7.80 (.10)**	8.14 (.12)	7.91 (.11)	7.85 (.12)	7.95 (.11)	8.17 (.13)	8.17 (.12)	7.98 (.14)	8.05 (.12)
Food-specific knowledge	**10.42 (.14)**	**10.31 (.13)**	**9.47 (.14)**	**9.33 (.13)**	**9.76 (.15)**	**10.11 (.14)**	**9.40 (.16)**	**9.31 (.14)**	**9.83 (.17)**	**10.01 (.16)**	**9.92 (.18)**	**9.44 (.15)**
Positive outcome expectations	2.91 (.03)	2.93 (.03)	2.86 (.03)	2.88 (.03)	2.85 (.03)	2.92 (.03)	2.92 (.04)	2.90 (.03)	2.95 (.04)	2.97 (.04)	2.97 (.04)	2.96 (.03)
FMFV preparation skills	**72.50 (.02)**	**72.19 (.82)**	**71.72 (.91)**	**69.63 (.84)**	73.06 (.98)	72.33 (.90)	72.53 (1.01)	71.55 (.97)	73.27 (1.09)	72.99 (1.01)	73.07 (1.13)	71.40 (.97)

*WFS* indicates WIC Fresh Start, *WFSV* WIC Fresh Start plus Farmers’ Market Nutrition Program vouchers, *EHE* online existing health education, *EHEV* online existing health education plus Farmers’ Market Nutrition Program vouchers, *FMNP* Farmers’ Market Nutrition Program, *FM* farmers’ market, *FV* fruit and vegetable(s), *LGSI* locally grown, seasonal items. Least square means and standard errors were derived from linear mixed effects models that controlled for baseline measures of each outcome and pregnancy status, breastfeeding status, receiving assistance other than WIC, and social desirability trait. Means highlighted in bold differ significantly from one another. Differences consistent with a priori hypotheses (WFSV > WFS, EHE, and EHEV) are reported. Knowledge of the FMNP: WFSV > EHE, EHEV (posttest); WFSV > WFS, EHE; WFS > EHEV (3-month follow-up); WFSV > WFS, EHE (6-month follow-up). FV food safety skills: WFSV > EHE, EHEV (posttest); WFSV > EHE, EHEV (3-month follow-up); WFS > EHE, EHEV (6-month follow-up). Farmers’ market-specific knowledge: WFSV > WFS, EHE (posttest); WFSV > WFS, EHE (3-month follow-up). Familiarity with locally grown, seasonal items: WFSV > EHEV (6-month follow-up). Knowledge of locally grown, seasonal items found at farmers’ markets in July: WFSV > EHEV (posttest). Food-specific knowledge: WFSV > EHE, EHEV (posttest); WFSV > WFS, EHE, EHEV (3-month follow-up); WFSV > EHEV (6-month follow-up). Farmers’ market FV preparation skills: WFSV > EHEV (posttest)

As shown in Table 4, significant differences by lesson (favoring those who received the WFS lesson as compared to EHE) were found in knowledge of the FMNP at posttest (*p* < .0001), FV food safety skills at posttest (*p* < .0001) and 3- and 6-month follow-up (*p* = .0001 and .0312, respectively), familiarity with locally grown seasonal items at posttest (*p* = .0062) and 6-month follow-up (*p* = .0217), knowledge of seasonal items found at farmers’ markets in July at posttest (*p* = .0077), food-specific knowledge at posttest (*p* < .0001) and 3-month follow-up (*p* < .0001), and farmers’ market FV preparation skills at posttest (*p* = .0293).

**Table 4:** Least square means and standard errors for secondary outcomes over time by lesson

	Least square means and standard errors					
	Posttest		3-month follow-up		6-month follow-up	
	WFS	EHE	WFS	EHE	WFS	EHE
Knowledge of the FMNP	**5.01 (.06)**	**4.69 (.06)**	4.96 (.06)	4.91 (.06)	4.93 (.07)	4.90 (.07)
Attitudes towards FMFV	11.53 (.47)	10.80 (.47)	10.96 (.51)	11.33 (.50)	10.98 (.56)	11.80 (.56)
FV food safety skills	**68.08 (.66)**	**63.65 (.66)**	**66.57 (.71)**	**63.20 (.71)**	**67.67 (.78)**	**65.54 (.78)**
FM-specific knowledge	1.55 (.06)	1.49 (.06)	1.61 (.06)	1.61 (.07)	1.62 (.07)	1.58 (.07)
FM asking skills	.82 (.21)	.87 (.23)	.72 (.24)	.86 (.30)	1.38 (.30)	1.60 (.36)
Familiarity with LGSI	**2.82 (.03)**	**2.69 (.03)**	2.83 (.04)	2.76 (.04)	**2.87 (.04)**	**2.74 (.04)**
Knowledge of LGSI found at FM in July	**8.15 (.08)**	**7.89 (.08)**	8.02 (.08)	7.91 (.08)	8.17 (.09)	8.02 (.09)
Food-specific knowledge	**10.36 (.10)**	**9.39 (.10)**	**9.93 (.11)**	**9.35 (.11)**	9.92 (.12)	9.65 (.12)
Positive outcome expectations	2.92 (.02)	2.88 (.02)	2.89 (.02)	2.91 (.02)	2.96 (.03)	2.97 (.03)
FMFV preparation skills	**72.25 (.66)**	**70.52 (.66)**	72.81 (3.58)	73.65 (3.56)	73.48 (4.01)	73.77 (3.99)

*WFS* indicates WIC Fresh Start, *EHE* online existing health education, *FM* farmers’ market, *FV* fruit and vegetable(s), *LGSI* locally grown, seasonal items. Least square means and standard errors were derived from linear mixed effects models that controlled for baseline measures of each outcome and pregnancy status, breastfeeding status, receiving assistance other than WIC, and social desirability trait. WFS and EHE means highlighted in bold differ significantly from one another

### Binary measures

As shown in Tables 5 and 6, significant differences by arm (favoring those in the WFSV arm as compared to the three other arms) were found in knowledge of WIC-authorized farmers’ markets at posttest (*p* < .0001) and 3- and 6-month follow-up (*p* = .0002 and .0006, respectively), ever having purchased FV at a farmers’ market at 3-month follow-up (*p* = .0003), and intentions to purchase FV at a farmers’ market at posttest (*p* < .0001) and 3-month follow-up (*p* = .0047). Significant lesson effects (favoring those who received the WFS lesson as compared to EHE) also were found in knowledge of WIC-authorized markets at posttest (*p* < .0001) and 3- and 6-month follow-up (*p* = .0021 and .0071, respectively) and intentions to purchase FV at a farmers’ market at posttest (*p* = .0076).

**Table 5:** Odds ratios and 95% confidence intervals for binary outcomes over time by trial arm

	Odds ratios, 95% confidence intervals, and *p* values											
	Posttest				3-month follow-up				6-month follow-up			
	Arm against which WFSV arm is compared				Arm against which WFSV arm is compared				Arm against which WFSV arm is compared			
Outcome	WFS	EHE	EHEV	*p*	WFS	EHE	EHEV	*p*	WFS	EHE	EHEV	*p*
Knowledge of WIC-authorized FM	1.85 (0.95, 3.58)	**4.51 (2.42, 8.42)**	**2.44 (1.29, 4.62)**	<.0001	**2.25 (1.04, 4.90)**	**5.06 (2.40, 10.69)**	**2.18 (1.01, 4.71)**	.0002	1.50 (0.64, 3.52)	**4.41 (2.00, 9.71)**	1.52 (0.68, 3.42)	.0006
Ever purchased FV at FM	1.78 (0.90, 3.53)	1.06 (0.52, 2.17)	1.31 (0.66, 2.58)	.2976	**8.70 (3.06, 25.00)**	**6.04 (2.06, 17.76)**	**3.36 (1.13, 10.04)**	.0003	1.65 (0.61, 4.48)	1.50 (0.53, 4.24)	1.05 (0.37, 2.96)	.6880
Purchased FV at FM past 2 weeks	1.07 (0.59, 1.96)	2.18 (1.14, 4.16)	1.25 (0.71, 2.21)	.0987	0.81 (0.39, 1.69)	2.23 (0.91, 5.47)	1.07 (0.53, 2.16)	.1760	0.44 (0.17, 1.13)	0.68 (0.25, 1.90)	0.78 (0.30, 2.04)	.3508
Intentions to purchase FV at FM	**2.92 (1.41, 6.02)**	**5.16 (2.57, 10.35)**	2.02 (0.97, 4.22)	<.0001	**2.04 (1.10, 3.80)**	**2.35 (1.25, 4.41)**	0.98 (0.52, 1.87)	.0047	0.97 (0.54, 1.77)	0.96 (0.52, 1.78)	1.09 (0.62, 1.92)	.9733

*WFSV* indicates WIC Fresh Start plus Farmers’ Market Nutrition Program vouchers, *WFS* WIC Fresh Start, *EHE* online existing health education, *EHEV* online existing health education plus Farmers’ Market Nutrition Program vouchers, *FM* farmers’ market, *FV* fruit and vegetable(s). Odds ratios and confidence intervals were derived from generalized linear mixed effects models that controlled for baseline measures of each outcome and pregnancy status, breastfeeding status, receiving assistance other than WIC, and social desirability trait. Odds ratios and confidence intervals indicate the likelihood of the outcome among those in the WFSV arm relative to the comparator. Values highlighted in bold indicate a significant difference between the comparator and participants in the WFSV arm. *P* values shown are for tests of the equality of odds ratios

**Table 6:** Odds ratios and 95% confidence intervals for binary outcomes over time by lesson

	Odds ratios, 95% confidence intervals, and *p* values					
	Posttest		3-month follow-up		6-month follow-up	
	WFS as compared to EHE lesson	*p*	WFS as compared to EHE lesson	*p*	WFS as compared to EHE lesson	*p*
Knowledge of WIC-authorized FM	**2.40 (1.59, 3.65)**	<.0001	**2.10 (1.31, 3.39)**	.0021	**2.08 (1.22, 3.57)**	.0071
Ever purchased FV at FM	0.86 (0.55, 1.37)	.5367	1.04 (0.61, 1.79)	.8872	0.89 (0.46, 1.76)	.7555
Purchased FV at FM past 2 weeks	1.53 (0.99, 2.35)	.0513	1.54 (0.90, 2.66)	.1159	1.15 (0.60, 2.11)	.6725
Intentions to purchase FV at FM	**1.78 (1.17, 2.74)**	.0076	1.01 (0.67, 1.53)	.9462	1.04 (0.69, 1.57)	.8380

*WFS* indicates WIC Fresh Start, *EHE* online existing health education, *FM* farmers’ market, *FV* fruit and vegetable(s). Odds ratios and confidence intervals were derived from generalized linear mixed effects models that controlled for baseline measures of each outcome and pregnancy status, breastfeeding status, receiving assistance other than WIC, and social desirability trait. Odds ratios and confidence intervals indicate the likelihood of the outcome among those completing the WFS lesson as compared to an EHE lesson. Values highlighted in bold indicate a significant difference by lesson. *P* values are for tests of the equality of odds ratios.

### Lesson effects on voucher redemption (hypotheses 3 and 4)

Only eight participants (1%) redeemed their CVV at farmers’ markets. Of these participants, seven received the WFS lesson and one received EHE (Fisher’s exact *p* < .05). Among FMNP voucher recipients, FMNP voucher redemption did not differ by lesson.

### Effect modification

Among all FMNP voucher recipients as well as in the subset of foreign-born recipients, Spanish language preference moderated lesson effects on FMNP voucher redemption (all *p* < .01 for the lesson by language preference interaction). There was a positive effect of the WFS lesson as compared to EHE on voucher redemption among those preferring to speak Spanish (OR = 2.08, 95% CI [1, 4.35]) whereas there was no lesson effect on voucher redemption among those not preferring to speak Spanish (OR = .97, 95% CI [.58, 1.60]). Moreover, while there was a strong positive effect of the WFS lesson as compared to EHE among foreign-born participants preferring to speak Spanish (OR = 2.36, 95% CI [1.06, 5.27]), there was no lesson effect among those not preferring to speak Spanish (OR = .33, 95% CI [.11, 1.01]). Among all FMNP voucher recipients, the FMNP voucher redemption rate was 44% among those who received the WFS lesson as compared to 38% among those who received EHE. Among foreign-born voucher recipients preferring to speak Spanish, the corresponding rates were 68% (WFS) and 48% (EHE).

## Discussion

This study examined primary and secondary outcomes associated with exposure to the WFS lesson relative to EHE among WIC-enrolled women who received FMNP vouchers and those who did not. Consistent with a priori hypotheses, exposure to the lesson was associated with FMNP voucher redemption (among FMNP voucher recipients preferring to speak Spanish), improvements in farmers’ market-related knowledge and skills and ever having and intentions to purchase FV at a farmers’ markets (among those who received the WFS lesson plus FMNP vouchers as compared to those who received the WFS lesson alone and EHE with and without FMNP vouchers), and modest gains in the redemption of CVV at farmers’ markets.

The lack of lesson effects on FV intake was surprising. This may be explained, in part, by participants’ higher-than-recommended fruit intake of 2.7 cups/day at baseline. Among women aged 19 to 30 years who get less than 30 min per day of moderate physical activity, 2 cups/day of fruit (including juice) are recommended; 1.5 cups/day are recommended among those aged 31 to 50 years [28]. There may have been little room for improvement in participants’ fruit intake and possibly a need for greater emphasis in the WFS lesson on the promotion of vegetable intake.

A strength of using an active control group in the WFS trial was the ability to control for attention, time, and expectations regarding the lesson received; a disadvantage, however, was that it may have been more difficult to detect an intervention effect [29]. It has been suggested that providing individuals with *any* active intervention content is likely to lead to some change in behavior [30]. Possibly, exposure to EHE decreased post-intervention between-group differences in FV intake, explaining the lack of condition effects found. Although the possibility that the lack of effects was due to the measures of FV intake was consdiered, this was unlikely for the following reasons: the measures had moderate validity and reliability, were administered according to standardized protocols, portion size measurement aids were used to facilitate the accurate reporting of amounts consumed of FV, and social desirability trait, shown to bias self-reported FV intake, was measured and controlled for in analyses [19–21, 27]. Alternatively, the lack of effects may be explained by insufficient lesson exposures. Most participants who received the WFS lesson did not open emails linking to follow-on content; as such, they did not receive this content as intended.

Improvements in targeted secondary outcomes were greatest among those in the WFSV arm as compared to the three other arms. For comparisons with EHE arms, effect sizes were large, approaching or exceeding one standard deviation at baseline for food-specific knowledge, and small to moderate (up to half of the standard deviation at baseline) for knowledge of the FMNP and FV food safety skills. These compare favorably with effect sizes reported for psychosocial outcome changes resulting from computer-mediated intervention [30]. Noted elsewhere, from a public health perspective, even modest changes are meaningful at the population level [30].

Lesson effects on the redemption of CVV at farmers’ markets are promising. Although modest, observed improvements suggest that CVV redemption should be a focus of future farmers’ market interventions to increase redemption rates. Although the option to redeem CVV at farmers’ markets was adopted in New Jersey nearly a decade ago, anecdotal evidence (observations by administrators and staff at the collaborating WIC agency) suggests that awareness of this option is limited. This may be because CVV are distributed year-round; as such, participants would need to be reminded that they can be redeemed at farmers’ markets at the beginning of the farmers’ market season. Strategies recommended for effectively reaching participants with this information, e.g., the development of a dissemination plan detailing when information will be provided and the use of such materials as leaflets or brochures accompanied by repetition from credible sources (for example, WIC nutritionists) should therefore be considered [31]. Intervention policies and programs to expand the reach of CVV also are needed. Accomplishing this will require strategies to overcome common barriers preventing states from adopting the option to allow the redemption of CVV at farmers’ markets, e.g., lack of resources to develop the required infrastructure; wanting to wait for the state to adopt electronic benefit transfer technology before implementing this option; concerns regarding the limited number of area markets and farmers’ capacity to provide sufficient amounts of food (and choices) to participants; and limited market hours, locations, and accessibility to the WIC population [32].

Lesson effects on FMNP voucher redemption in the subset of voucher recipients preferring to speak Spanish were unexpected. Among foreign-born Hispanics, language preference has been used as an indicator of acculturation, with individuals preferring to speak Spanish considered less acculturated than those preferring to speak English [33]. The strongest effects were found among FMNP voucher recipients with low language acculturation. Previous work has shown that acculturation is associated with unhealthy food consumption behaviors, including low FV intake [34–36]. Farmers’ market use is found to be higher among foreign- as compared to US-born Hispanic mothers [37]. In research with foreign-born Hispanic women, cultural preferences for fresh FV have been identified [38–40]. In one study, participants reported using FMNP vouchers, local produce from friends and neighbors, and produce grown by families themselves to acquire fresh FV they desired [38]. In another, fresh FV were considered better suited to cooking styles and beliefs about how meals should be prepared; the preparation of traditional dishes requiring fresh vegetables was common [39]. In a third study, healthy food was defined based on such qualities as freshness (as indicated by time since harvest), purity (as indicated by the absence of preservatives and processing) and naturalness (as indicated by chemical-free farming practices) [40]. The healthier diets, higher rate of farmers’ market use, and preferences for fresh FV found among foreign-born and less acculturated Hispanics suggests that those with low language acculturation may have been more receptive to the WFS lesson and correspondingly, more likely to shop at farmers’ markets and to redeem their FMNP vouchers.

The self-selected sample is a limitation of the study. Although a representative sample would have strengthened the generalizability of findings, probability sampling was not possible in light of time and cost constraints of the study. Transportation issues (lack of access to transportation and distance to markets) were a prominent barrier to farmers’ market use identified in initial research with representatives of the target population. Addressing this barrier was considered beyond the scope of the work. Social Cognitive Theory emphasizes intervening to improve both the food environment (e.g., FV access) and behavioral capacity (knowledge and skills to increase FV intake) [41, 42]. As such, a multilevel approach to comprehensively address influences on farmers’ market use may have improved lesson effectiveness. The randomized longitudinal design, relatively large sample size, active control group, validated measures of FV intake, and objective measures of FMNP voucher and CVV redemption are study strengths.

## Conclusion

Theory-driven, web-based nutrition education is promising for promoting FMNP voucher redemption, farmers’ market-related knowledge and skills, ever having purchased and intentions to purchase FV at farmers’ markets, and the redemption of CVV at farmers’ among WIC-enrolled women. Further research is needed to explore factors that may explain the lack of intervention effects on FV intake. Studies of whether the effects differ based on pre-intervention levels of intake and outcomes associated with a stronger focus on vegetable intake in the lesson are needed. There is also a need to identify optimal approaches for improving the uptake of follow-on content. Warranting investigation is the utility of reminders (sent via mail and text messaging) found effective in improving uptake of health interventions in hard to reach communities [43, 44]. Text messaging may be particularly effective as it is among the communication technologies most often used by WIC participants and is recommended for reinforcing WIC nutrition education [45, 46].

## Abbreviations

CVV: Cash value voucher(s)
EHE: Online existing health education
EHEV: Online existing health education plus FMNP vouchers
FMNP: Farmers’ Market Nutrition Program
FV: Fruit and vegetable(s)
WFS: WIC Fresh Start
WFSV: WIC Fresh Start plus FMNP vouchers
WIC: Special Supplemental Nutrition Program for Women, Infants and Children
